# Nonlinear Association Between the C‐Reactive Protein‐To‐Albumin Ratio and Post‐Stroke Epilepsy Risk

**DOI:** 10.1002/cns.70967

**Published:** 2026-07-16

**Authors:** Xiao Wu, Yingru Zhou, Qi Yang, Yunliang Tang

**Affiliations:** ^1^ Department of Neurosurgery Jiangxi Key Laboratory of Neurological Diseases, the First Affiliated Hospital, Jiangxi Medical College, Nanchang University Nanchang Jiangxi China; ^2^ Department of Rehabilitation Medicine The First Affiliated Hospital, Jiangxi Medical College, Nanchang University Nanchang China

**Keywords:** C‐reactive protein‐to‐albumin ratio, ischemic stroke, post‐stroke epilepsy, predictor, retrospective cohort study

## Abstract

**Background:**

The C‐reactive protein‐to‐albumin ratio (CAR) is an integrated biomarker of inflammation and nutritional status. Its potential association with the risk of post‐stroke epilepsy (PSE) after ischemic stroke (IS) requires comprehensive evaluation.

**Methods:**

We analyzed data from 21,459 IS patients admitted to hospitals in Chongqing, China, between June 2017 and July 2023. CAR was calculated from admission laboratory values. The primary outcome was the development of PSE within 1 year. Multivariable logistic regression with three progressively adjusted models was used to control for demographic factors, stroke severity (NIHSS), comorbidities, neuroimaging findings, and extensive laboratory parameters. A restricted cubic spline (RCS) analysis explored the relationship's nonlinearity. Subgroup and sensitivity analyses tested robustness.

**Results:**

Elevated admission CAR was independently associated with increased PSE risk. After full adjustment, each unit increase in CAR yielded an odds ratio (OR) of 1.88 (95% CI: 1.64–2.16, *p* < 0.001). The ROC analysis showed that the AUC for CAR in predicting PSE was 0.84 (95% CI: 0.83–0.85). RCS analysis revealed a significant nonlinear relationship (p‐nonlinearity < 0.001) with an inflection point at CAR = 1.15. A pronounced dose–response relationship was observed across CAR quartiles, with the highest quartile (Q4) showing a substantially elevated risk (adjusted OR = 34.42, 95% CI: 18.58–69.70) compared to the lowest (Q1). Subgroup analyses indicated particularly strong associations in patients with diabetes, coronary artery disease, and middle cerebral artery involvement, confirmed by sensitivity analyses.

**Conclusion:**

CAR is a potent, independent predictor of PSE in IS patients, demonstrating a nonlinear, threshold‐based relationship. As an integrative marker, it may enhance early risk stratification and guide personalized interventions, warranting further prospective validation.

## Graphical abstract

The C‐reactive protein‐to‐albumin ratio (CAR) is a robust, independent predictor of post‐stroke epilepsy (PSE) in ischemic stroke patients, exhibiting a nonlinear, threshold‐dependent association. As an integrative biomarker, CAR holds promise for enhancing early risk stratification and informing personalized intervention strategies.

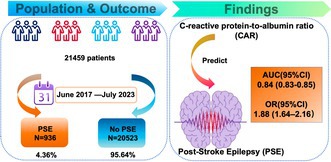

## 1 Introduction

Stroke is a leading cause of disability and mortality worldwide, yet effective treatments remain elusive [1, 2, 3]. Ischemic stroke (IS) is the predominant form, comprising approximately 80% of all stroke cases [4]. Post‐stroke epilepsy (PSE), a significant complication following stroke, affects about 5% to 10% of stroke survivors [5, 6], with stroke being responsible for nearly half of new‐onset epilepsy cases in the elderly population [7, 8]. PSE is associated with poorer clinical outcomes [9, 10, 11], and individuals with PSE have a mortality rate approximately twice as high 1 year post‐stroke compared to those without PSE. Therefore, identifying accurate early predictors of PSE is essential for the development of personalized management strategies for stroke survivors.

While PSE is a complex neurological disorder, electroencephalography (EEG) remains one of the most well‐established and accessible biomarkers for the disease. Nevertheless, the prognostic value of early EEG findings in predicting PSE has not been thoroughly investigated. Current predictors predominantly encompass clinical and neuroimaging parameters, such as acute symptomatic seizures occurring within 7 days post‐stroke, seizure type and timing [12, 13], stroke severity, large‐artery atherosclerosis, cortical involvement, middle cerebral artery (MCA) territory involvement, and hemorrhagic conversion [14, 15]. Additionally, stroke interventions, including decompressive craniectomy, craniotomy, intravenous alteplase, and endovascular treatment, are recognized as risk factors for PSE. These variables are integrated into clinical scoring systems, such as the CAVE and SeLECT models [16, 17]. However, nearly half of these models have not undergone external validation, and the accuracy of PSE prediction remains suboptimal.

The inflammatory response is implicated in the etiology of various neurological disorders and plays a crucial role in the progression and prognosis of these diseases. Recent evidence suggests that neuroinflammation acts both as a cause and a consequence of disease in patients with epilepsy. Inflammatory intermediates, including interleukins, tumor necrosis factor α, nuclear factor‐kappa B, and high‐mobility group box 1 protein, are expressed, and toll‐like receptors are upregulated in animal models of epilepsy [18]. Activated microglia, reactive astrocytes, and inflammatory intermediates may contribute to hyperexcitability in seizure foci [19]. Both C‐reactive protein (CRP) and albumin are synthesized by the liver as part of the acute inflammatory response. The CRP to albumin ratio (CAR) thus integrates the pro‐inflammatory and nutritional status of patients into a single index, potentially providing superior prognostic information compared to either parameter alone. Recent studies have indicated that the CAR offers a more comprehensive understanding of the inflammatory response [20]. Nonetheless, the role of CAR as a risk indicator in PSE remains an underexplored area.

The aim of this study was to examine the relationship between the CAR and the risk of PSE in individuals with IS, as well as to evaluate its potential utility in risk stratification. Understanding this association could offer significant insights into the mechanisms underlying PSE and enable timely interventions for individuals at risk. Furthermore, the results may inform the development of more targeted preventive strategies, thereby reducing the public health burden associated with IS.

## 2 Methods

### 2.1 Data Source and Study Population

The data were obtained from the Dryad Digital Repository database, which is an open‐resource database that provides a broad range of discoverable, freely reusable, reference research data. All authors have waived their copyright to these original research data. The dataset used in this study was shared by Liu et al. [21]. In short, this retrospective study utilized data from four hospitals located in Chongqing, China. Patient records were obtained from the Chongqing Emergency Center for the period spanning June 2017 to June 2022, and from Qianjiang Central Hospital, Bishan District People's Hospital, and Yubei District Traditional Chinese Medicine Hospital from July 2022 to July 2023. The inclusion criteria for the study were as follows: (1) patients aged between 18 and 90 years at the time of admission; (2) a diagnosis of acute ischemic stroke followed by hospitalization for treatment. The exclusion criteria included: (1) a history of previous stroke or transient ischemic attack (TIA); (2) a history of traumatic brain injury, intracranial tumors, or cerebral vascular malformations that could predispose individuals to epilepsy; (3) a prior diagnosis of epilepsy or the use of antiseizure medications for any reason, such as seizure prophylaxis, migraine, or psychiatric disorders; (4) mortality occurring within 72 h following the onset of stroke.

### 2.2 Data Collection

Data for this study were systematically collected through comprehensive review of patient electronic health records. The collected variables encompassed key patient demographics (Age, Gender), detailed comorbidities (Uremia, Deep Vein Thrombosis [DVT], Fatty liver disease/Steatosis, Diabetes mellitus, Hypertension, Coronary Artery Disease [CAD], Atrial Fibrillation, Cerebral Herniation, Hydrocephalus, Hyperuricemia, Hyperlipidaemia, Hypoproteinemia), specific neuroanatomical localization of pathologies (Frontal Lobe, Parietal Lobe, Temporal Lobe, Occipital Lobe, Insular Lobe, Basal Ganglia, Capsula Interna, Brainstem, Epencephalon, Paraventricular region, Centrum Semiovale, Thalamus, Subcortical Lobe), vascular territories involved (Anterior Cerebral Artery [ACA], Middle Cerebral Artery [MCA], Posterior Cerebral Artery [PCA], Vertebral Artery [VA], Basilar Artery), cerebral circulatory anatomy (Anterior Circle, Posterior Circle), plaque presence and location (Common Carotid Artery [CCA] plaque, Internal Carotid Artery [ICA] plaque, External Carotid Artery [ECA] plaque), classification of Large Vessel Disease, and extensive laboratory parameters including hematology (Platelet Count [PLT], White Blood Cell Count [WBC], Red Blood Cell Count [RBC]), metabolic markers (Glycated Hemoglobin [HbA1c], C‐Reactive Protein [CRP], Triglycerides [TG], Low‐Density Lipoprotein [LDL‐c], High‐Density Lipoprotein [HDL‐c], Lactate), liver function tests (Aspartate Aminotransferase [AST], Alanine Aminotransferase [ALT], Bilirubin, Albumin), renal function tests (Urea, Creatinine, Uric Acid [UA]), and coagulation profiles (D‐Dimer, Fibrinogen).

### 2.3 Outcome Definition

The primary outcome was the development of PSE within 1 year after the acute IS. Diagnoses of acute IS were based on the Chinese Guidelines for the Diagnosis and Treatment of Acute Ischemic Stroke [22]. PSE is defined as newly developed, secondary epilepsy occurring after an acute ischemic stroke in patients with no prior history of epilepsy or antiseizure medication use. Patients whose seizures might have been caused by other factors (such as brain tumors, intracranial vascular malformations, or traumatic brain injury) were excluded [21, 23].

### 2.4 Calculation of CAR

The CAR was calculated using the following formula: CAR = CRP (mg/L) / Albumin (g/L).

### 2.5 Statistical Analysis

Descriptive statistics were employed to summarize the baseline demographic and clinical characteristics of the study cohort. Continuous variables were expressed as means with standard deviations (SD), while categorical variables were represented as counts and percentages. Group comparisons were conducted using one‐way analysis of variance (ANOVA). Categorical variables were presented as frequencies and percentages and analyzed using chi‐square tests. Logistic regression analyses were utilized to assess the associations between the CAR and PSE, with adjustments made for potential confounders. Three progressively adjusted models were developed: Model 1 was unadjusted; Model 2 included adjustments for age, gender, NIHSS, uremia, DVT, fatty liver, diabetes, hypertension, CAD, atrial fibrillation, and cerebral herniation; and Model 3 further incorporated anterior circle, posterior circle, large vessel disease, PLT, WBC, RBC, HbA1c, TG, HDL‐C, LDL‐C, and creatinine. Both continuous and categorical forms of CAR were analyzed, with odds ratios (ORs), 95% confidence intervals (CIs), and *p*‐values reported. Restricted cubic spline (RCS) analysis was applied to evaluate the shape of the relationship between CAR and PSE. The Likelihood Ratio Test was employed to assess nonlinear associations, with knots strategically positioned at the 5th, 35th, 65th, and 95th percentiles of the CAR distribution, using the median value as a reference point. All RCS models were adjusted for the same covariates as those in logistic regression Model 3. Stratified subgroup analyses were conducted based on variables such as the presence of DVT, diabetes, CAD, and the involvement of specific neuroanatomical structures, including the basal ganglia, paraventricular region, centrum semiovale, middle cerebral artery territory, common carotid artery plaque, subcortical lobe, and anterior or posterior circulations. Interaction terms between CAR, treated as a continuous variable, and each subgroup variable were incorporated into logistic regression models to evaluate potential effect modification. All subgroup models were adjusted for the same set of covariates as in Model 3, excluding the stratification variable itself to prevent over‐adjustment. Data processing and statistical analyses were performed using R software (version 4.5.0). A two‐sided *p*‐value of less than 0.05 was considered indicative of statistical significance.

## 3 Results

### 3.1 Baseline Characteristics

The baseline characteristics of the study participants, categorized by CAR quartiles, are detailed in Table 1. Statistically significant differences were identified across most of the measured variables among the four CAR groups (all *p* < 0.001). Participants in the higher CAR quartiles (Q3 and Q4) were generally older and exhibited higher NIHSS scores, suggesting increased stroke severity. Additionally, these groups demonstrated a more adverse inflammatory and metabolic profile, as evidenced by significantly elevated levels of WBC, HbA1c, D‐dimer, and fibrinogen, coupled with decreased levels of RBC and PLT. The prevalence of complications, including large vessel disease, DVT, diabetes, atrial fibrillation, hydrocephalus, and hypoproteinemia, was also significantly higher in the upper CAR quartiles compared to the lowest quartile (Q1).

**TABLE 1:** Baseline characteristics stratified by CAR quartiles.

Variable		Q1 (*n* = 5365)	Q2 (*n* = 5365)	Q3 (*n* = 5364)	Q4 (*n* = 5365)	*p*
Age, years		58.64 ± 10.34	65.21 ± 10.53	69.28 ± 12.03	72.52 ± 11.85	< 0.001
Gender, %	male	3476 (64.8)	2466 (46.0)	2218 (41.3)	2456 (45.8)	< 0.001
	female	1889 (35.2)	2899 (54.0)	3146 (58.7)	2909 (54.2)	
NIHSS, %	< 5	671 (12.5)	536 (10.0)	228 (4.3)	94 (1.8)	< 0.001
	5–14	4587 (85.5)	4792 (89.3)	5056 (94.3)	4936 (92.0)	
	> 14	107 (2.0)	37 (0.7)	80 (1.5)	335 (6.2)	
Large vessel disease, %	no	4582 (85.4)	3589 (66.9)	3606 (67.2)	4177 (77.9)	< 0.001
	yes	783 (14.6)	1776 (33.1)	1758 (32.8)	1188 (22.1)	
PLT, ×10^9/L (cells/L)		193.51 ± 27.65	192.24 ± 27.24	188.93 ± 24.90	184.91 ± 27.39	< 0.001
WBC, ×10^9/L (cells/L)		7.85 ± 1.32	8.09 ± 1.18	8.55 ± 1.35	9.41 ± 1.91	< 0.001
RBC, ×10^12/L (cells/L)		4.30 ± 0.33	4.38 ± 0.31	4.34 ± 0.32	4.23 ± 0.32	< 0.001
HbA1c, %		6.22 ± 0.84	6.69 ± 0.78	6.81 ± 0.86	6.96 ± 1.03	< 0.001
CRP, mg/L		3.09 ± 1.00	6.87 ± 1.33	13.43 ± 2.77	44.73 ± 31.07	< 0.001
TG, mmol/L		1.55 ± 0.49	1.65 ± 0.44	1.53 ± 0.38	1.45 ± 0.39	< 0.001
LDL‐c, mmol/L		2.72 ± 0.42	2.71 ± 0.30	2.68 ± 0.33	2.63 ± 0.38	< 0.001
HDL‐c, mmol/L		1.27 ± 0.18	1.23 ± 0.14	1.25 ± 0.14	1.25 ± 0.14	< 0.001
AST, U/L		22.45 ± 4.81	23.38 ± 4.51	25.45 ± 6.20	35.11 ± 23.13	< 0.001
ALT, U/L		22.84 ± 5.98	23.12 ± 5.29	22.54 ± 5.46	29.03 ± 17.02	< 0.001
Bilirubin, μmol/L		14.47 ± 4.52	14.55 ± 3.58	15.06 ± 3.26	16.95 ± 4.02	< 0.001
Albumin, g/L		42.30 ± 1.82	41.46 ± 1.45	40.77 ± 1.76	39.12 ± 2.49	< 0.001
Urea, mmol/L		6.12 ± 1.71	6.17 ± 1.17	6.53 ± 1.10	6.80 ± 1.55	< 0.001
Creatinine, μmol/L		69.68 ± 30.89	85.33 ± 61.36	92.99 ± 61.44	91.53 ± 40.48	< 0.001
UA, μmol/L		324.24 ± 48.53	356.71 ± 60.94	360.34 ± 54.18	335.27 ± 62.88	< 0.001
D‐dimer, ng/mL		0.71 ± 0.71	0.99 ± 0.65	1.24 ± 0.80	2.35 ± 2.32	< 0.001
Fibrinogen, g/L		3.23 ± 0.36	3.53 ± 0.34	3.70 ± 0.35	3.93 ± 0.53	< 0.001
Uremia (%)	no	5339 (99.5)	5313 (99.0)	5313 (99.0)	5304 (98.9)	0.002
	yes	26 (0.5)	52 (1.0)	51 (1.0)	61 (1.1)	
DVT (%)	no	5291 (98.6)	5172 (96.4)	5011 (93.4)	4661 (86.9)	< 0.001
	yes	74 (1.4)	193 (3.6)	353 (6.6)	704 (13.1)	
Fatty liver disease or steatosis (%)	no	4232 (78.9)	3967 (73.9)	4345 (81.0)	4670 (87.0)	< 0.001
	yes	1133 (21.1)	1398 (26.1)	1019 (19.0)	695 (13.0)	
Diabetes (%)	no	4485 (83.6)	3356 (62.6)	3107 (57.9)	3189 (59.4)	< 0.001
	yes	880 (16.4)	2009 (37.4)	2257 (42.1)	2176 (40.6)	
Hypertension (%)	no	2307 (43.0)	1203 (22.4)	1316 (24.5)	1882 (35.1)	< 0.001
	yes	3058 (57.0)	4162 (77.6)	4048 (75.5)	3483 (64.9)	
CAD (%)	no	3545 (66.1)	2699 (50.3)	2567 (47.9)	2976 (55.5)	< 0.001
	yes	1820 (33.9)	2666 (49.7)	2797 (52.1)	2389 (44.5)	
Atrial fibrillation (%)	no	5143 (95.9)	4874 (90.8)	4612 (86.0)	4787 (89.2)	< 0.001
	yes	222 (4.1)	491 (9.2)	752 (14.0)	578 (10.8)	
Cerebral herniation (%)	no	5350 (99.7)	5353 (99.8)	5292 (98.7)	5283 (98.5)	< 0.001
	yes	15 (0.3)	12 (0.2)	72 (1.3)	82 (1.5)	
Hydrocephalus (%)	no	5327 (99.3)	5320 (99.2)	5306 (98.9)	5225 (97.4)	< 0.001
	yes	38 (0.7)	45 (0.8)	58 (1.1)	140 (2.6)	
Hyperuricemia (%)	no	5247 (97.8)	4500 (83.9)	4476 (83.4)	4889 (91.1)	< 0.001
	yes	118 (2.2)	865 (16.1)	888 (16.6)	476 (8.9)	
Hyperlipidaemia (%)	no	3977 (74.1)	3975 (74.1)	4439 (82.8)	4599 (85.7)	< 0.001
	yes	1388 (25.9)	1390 (25.9)	925 (17.2)	766 (14.3)	
Hypoproteinemia (%)	no	5335 (99.4)	5351 (99.7)	4905 (91.4)	3447 (64.2)	< 0.001
	yes	30 (0.6)	14 (0.3)	459 (8.6)	1918 (35.8)	
Frontal lobe (%)	no	5277 (98.4)	5212 (97.1)	5015 (93.5)	5036 (93.9)	< 0.001
	yes	88 (1.6)	153 (2.9)	349 (6.5)	329 (6.1)	
Parietal lobe (%)	no	5307 (98.9)	5264 (98.1)	5125 (95.5)	5100 (95.1)	< 0.001
	yes	58 (1.1)	101 (1.9)	239 (4.5)	265 (4.9)	
Temporal lobe (%)	no	5296 (98.7)	5265 (98.1)	5131 (95.7)	5131 (95.6)	< 0.001
	yes	69 (1.3)	100 (1.9)	233 (4.3)	234 (4.4)	
Occipital lobe (%)	no	5332 (99.4)	5303 (98.8)	5231 (97.5)	5198 (96.9)	< 0.001
	yes	33 (0.6)	62 (1.2)	133 (2.5)	167 (3.1)	
Insular lobe (%)	no	5327 (99.3)	5334 (99.4)	5251 (97.9)	5268 (98.2)	< 0.001
	yes	38 (0.7)	31 (0.6)	113 (2.1)	97 (1.8)	
Basal ganglia (%)	no	5176 (96.5)	5119 (95.4)	5070 (94.5)	5126 (95.5)	< 0.001
	yes	189 (3.5)	246 (4.6)	294 (5.5)	239 (4.5)	
Capsula interna (%)	no	5361 (99.9)	5361 (99.9)	5360 (99.9)	5363 (100.0)	0.836
	yes	4 (0.1)	4 (0.1)	4 (0.1)	2 (0.0)	
Brainstem (%)	no	5347 (99.7)	5303 (98.8)	5283 (98.5)	5254 (97.9)	< 0.001
	yes	18 (0.3)	62 (1.2)	81 (1.5)	111 (2.1)	
Epencephalon (%)	no	5332 (99.4)	5282 (98.5)	5218 (97.3)	5185 (96.6)	< 0.001
	yes	33 (0.6)	83 (1.5)	146 (2.7)	180 (3.4)	
Paraventricular (%)	no	5232 (97.5)	5039 (93.9)	5033 (93.8)	5108 (95.2)	< 0.001
	yes	133 (2.5)	326 (6.1)	331 (6.2)	257 (4.8)	
Centrum semiovale (%)	no	5350 (99.7)	5149 (96.0)	5151 (96.0)	5199 (96.9)	< 0.001
	yes	15 (0.3)	216 (4.0)	213 (4.0)	166 (3.1)	
Thalamus (%)	no	5312 (99.0)	5306 (98.9)	5304 (98.9)	5300 (98.8)	0.743
	yes	53 (1.0)	59 (1.1)	60 (1.1)	65 (1.2)	
Anterior cerebral artery (%)	no	5350 (99.7)	5328 (99.3)	5251 (97.9)	5256 (98.0)	< 0.001
	yes	15 (0.3)	37 (0.7)	113 (2.1)	109 (2.0)	
Middle cerebral artery (%)	no	5254 (97.9)	5140 (95.8)	5068 (94.5)	5169 (96.3)	< 0.001
	yes	111 (2.1)	225 (4.2)	296 (5.5)	196 (3.7)	
Posterior cerebral artery (%)	no	5363 (100.0)	5364 (100.0)	5340 (99.6)	5330 (99.3)	< 0.001
	yes	2 (0.0)	1 (0.0)	24 (0.4)	35 (0.7)	
Vertebral artery (%)	no	5301 (98.8)	5227 (97.4)	5128 (95.6)	5151 (96.0)	< 0.001
	yes	64 (1.2)	138 (2.6)	236 (4.4)	214 (4.0)	
Basilar artery (%)	no	5358 (99.9)	5319 (99.1)	5286 (98.5)	5305 (98.9)	< 0.001
	yes	7 (0.1)	46 (0.9)	78 (1.5)	60 (1.1)	
CCA plaque (%)	no	4686 (87.3)	3823 (71.3)	3818 (71.2)	4309 (80.3)	< 0.001
	yes	679 (12.7)	1542 (28.7)	1546 (28.8)	1056 (19.7)	
ICA plaque (%)	no	5204 (97.0)	4889 (91.1)	4858 (90.6)	5046 (94.1)	< 0.001
	yes	161 (3.0)	476 (8.9)	506 (9.4)	319 (5.9)	
ECA plaque (%)	no	5354 (99.8)	5328 (99.3)	5281 (98.5)	5295 (98.7)	< 0.001
	yes	11 (0.2)	37 (0.7)	83 (1.5)	70 (1.3)	
Subcortical lobe (%)	no	5022 (93.6)	4708 (87.8)	4605 (85.9)	4681 (87.3)	< 0.001
	yes	343 (6.4)	657 (12.2)	759 (14.1)	684 (12.7)	
Anterior circle (%)	no	5151 (96.0)	4981 (92.8)	4893 (91.2)	5021 (93.6)	< 0.001
	yes	214 (4.0)	384 (7.2)	471 (8.8)	344 (6.4)	
Posterior circle (%)	no	4073 (75.9)	4123 (76.8)	4286 (79.9)	4476 (83.4)	< 0.001
	yes	1292 (24.1)	1242 (23.2)	1078 (20.1)	889 (16.6)	

*Note:* Values are expressed as number (percentage) or mean ± SD.

Abbreviations: ACA, Anterior cerebral artery; ALT, alanine aminotransferase; AST, aspartate aminotransferase; BA, Basilar artery; CK, creatine kinase; CK‐MB, creatine kinase MB; CRP, C‐reactive protein; DVT, deep vein thrombosis; HBDH, hydroxybutyrate dehydrogenase; HDL‐c, High‐density lipoprotein; INR, International normalized ratio; LDH, lactate dehydrogenase; LDL‐c, Low‐density lipoprotein; MCA, Middle cerebral artery; PCA, Posterior cerebral artery; PLT, platelet count; PT, prothrombin time; RBC, red blood cell count; TG, triglycerides; TT, thrombin time; UA, blood uric acid; VA, Vertebral artery; WBC, white blood cell count.

### 3.2 Association of CAR With PSE Risk in IS Patients

Among the 21,459 patients included in the analysis, 936 patients developed PSE within 1 year, resulting in an incidence rate of approximately 4.36%. The relationship between CAR and the incidence of PSE is comprehensively presented in Table 2. In the unadjusted model (Model 1), each 1‐unit increase in CAR was significantly associated with increased odds of PSE (OR: 3.36; 95% CI: 3.14–3.59). This positive association persisted after successive adjustments for demographic and clinical confounders in Model 2 (OR: 5.24; 95% CI: 4.77–5.76) and further adjustments for laboratory and imaging parameters in Model 3 (adjusted OR: 1.88; 95% CI: 1.64–2.16), with all *p*‐values < 0.001. Analysis by quartiles revealed a clear dose–response relationship. Using the first quartile (Q1) as a reference, the adjusted odds ratios (Model 3) for PSE increased significantly across quartiles: Q2 (OR: 1.81; 95% CI: 0.86–4.00; *p* = 0.125), Q3 (OR: 13.74; 95% CI: 7.32–28.11; *p* < 0.001), and Q4 (OR: 34.42; 95% CI: 18.58–69.70; *p* < 0.001), indicating that patients in the highest CAR quartile exhibited a substantially elevated risk of developing PSE.

**TABLE 2:** Association between the CAR and PSE incidence in patients with acute ischemic stroke.

Characteristics	Case/total, *n*	Model 1 OR (95% CI)	*p*	Model 2 OR (95% CI)	*p*	Model 3 OR (95% CI)	*p*
CAR (per 1 unit)	936/21459	3.36 (3.14–3.59)	< 0.001	5.24 (4.77–5.76)	< 0.001	1.88 (1.64–2.16)	< 0.001
CAR quartile							
Quartile 1	20/5365	Reference		Reference		Reference	
Quartile 2	26/5365	1.30 (0.73–2.36)	0.377	1.70 (0.95–3.11)	0.076	1.81 (0.86–4.00)	0.125
Quartile 3	166/5364	8.53 (5.50–14.02)	< 0.001	12.58 (8.02–20.85)	< 0.001	13.74 (7.32–28.11)	< 0.001
Quartile 4	724/5365	41.69 (27.47–67.34)	< 0.001	75.59 (49.00–123.64)	< 0.001	34.42 (18.58–69.70)	< 0.001

*Note:* Model 1: unadjusted for any covariates, Model 2: adjusted for age, gender, NIHSS, uremia, DVT, fatty liver, diabetes, hypertension, CAD, atrial fibrillation, and cerebral herniation, Model 3: Model 2 + further adjusted for anterior circle, posterior circle, large vessel disease, PLT, WBC, RBC, HbA1c, TG, HDL‐C, LDL‐C, creatinine.

### 3.3 Nonlinear Association Analysis

**FIGURE 1:**
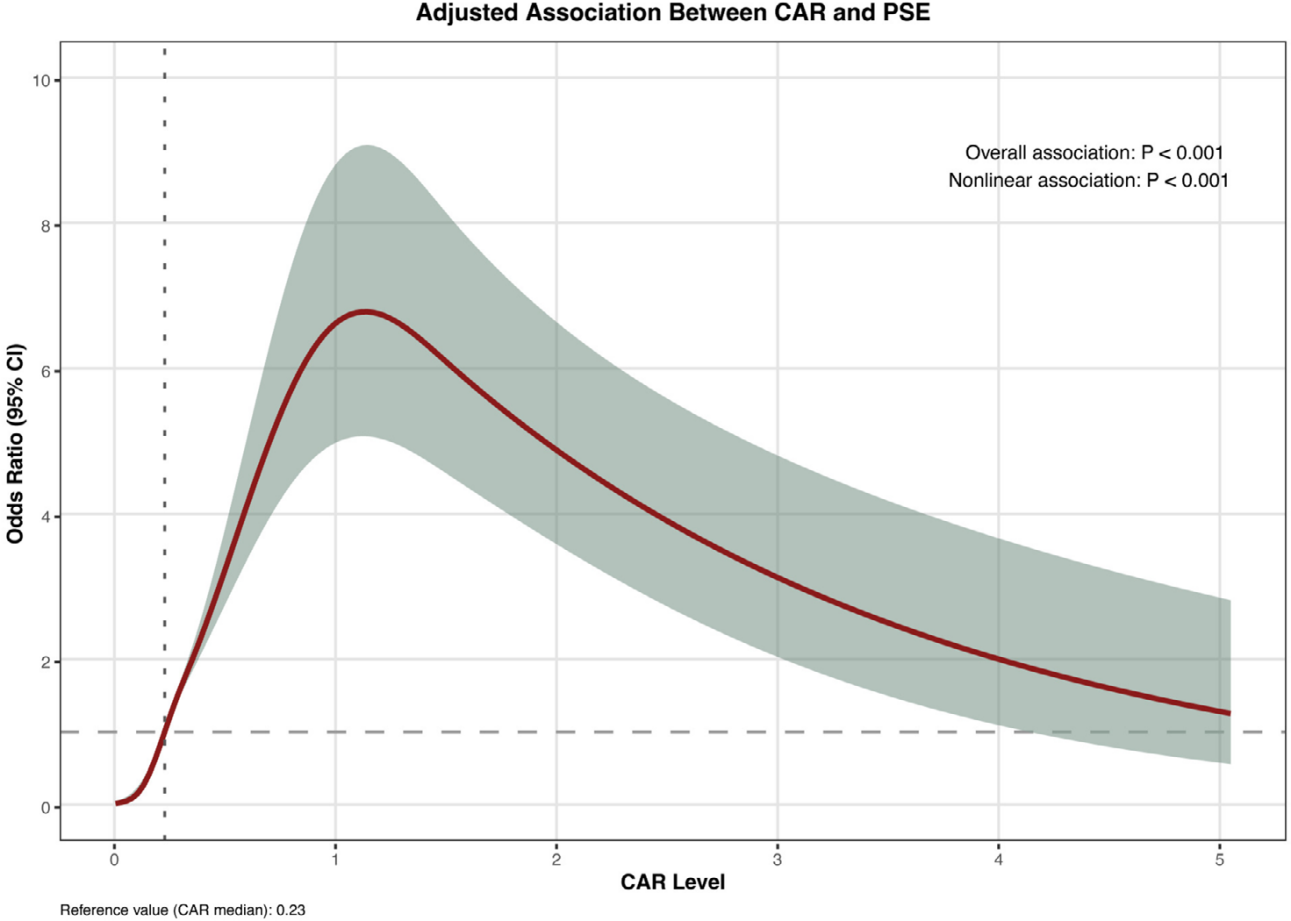
The dose–response relationship between CAR and the risk of PSE in patients with ischemic stroke, analyzed using multivariable‐adjusted restricted cubic spline (RCS) regression. The model was adjusted for various covariates, including age, gender, NIHSS, uremia, DVT, fatty liver, diabetes, hypertension, CAD, atrial fibrillation, cerebral herniation, anterior and posterior circulation, large vessel disease, PLT, WBC, RBC, HbA1c, TG, HDL‐C, LDL‐C, and creatinine.

**FIGURE 2:**
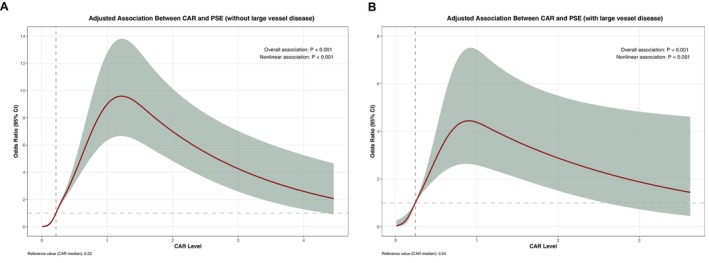
RCS analysis of the association between CAR and PSE incidence in ischemic stroke patients, stratified by large vessel disease: (A) the no large vessel disease group and (B) the large vessel disease group. Both models were adjusted for the same covariates as mentioned above.

**FIGURE 3:**
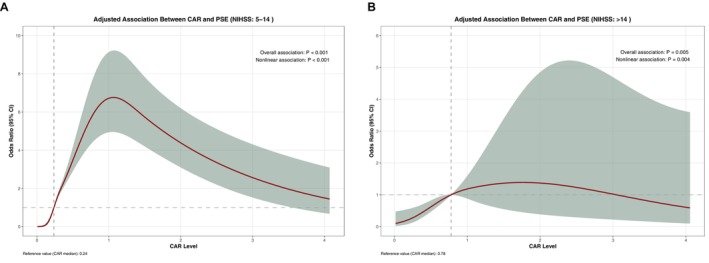
RCS analysis of CAR and PSE incidence in ischemic stroke patients, stratified by NIHSS score: (A) NIHSS (5–14) group and (B) NIHSS (> 14) group. Both models were similarly adjusted for age, gender, uremia, DVT, fatty liver, diabetes, hypertension, CAD, atrial fibrillation, cerebral herniation, anterior and posterior circulation, large vessel disease, PLT, WBC, RBC, HbA1c, TG, HDL‐C, LDL‐C, and creatinine.

RCS regression with multivariable‐adjusted associations was employed to illustrate dose–response relationships between the CAR and the prevalence of PSE across five distinct groups: the entire population, the large vessel disease group, the no large vessel disease group, the NIHSS (5–14) group, and the NIHSS (> 14) group. In the overall population, an inflection point was identified at CAR = 1.15. Figure 1 demonstrates a significant nonlinear association between CAR and PSE risk among participants with ischemic stroke (IS) (*p* < 0.001 and P for nonlinearity < 0.001). In the no large vessel disease group, Figure 2A indicates a significant nonlinear relationship between CAR and PSE risk among IS participants (*p* < 0.001 and P for nonlinearity < 0.001). Similarly, in the large vessel disease group, Figure 2B shows a significant nonlinear association (*p* < 0.001 and P for nonlinearity < 0.001). For the NIHSS (5–14) group, Figure 3A reveals a significant nonlinear relationship (*p* < 0.001 and P for nonlinearity < 0.001), whereas for the NIHSS (> 14) group, Figure 3B indicates a significant nonlinear association (*p* = 0.005 and P for nonlinearity = 0.004).

**FIGURE 4:**
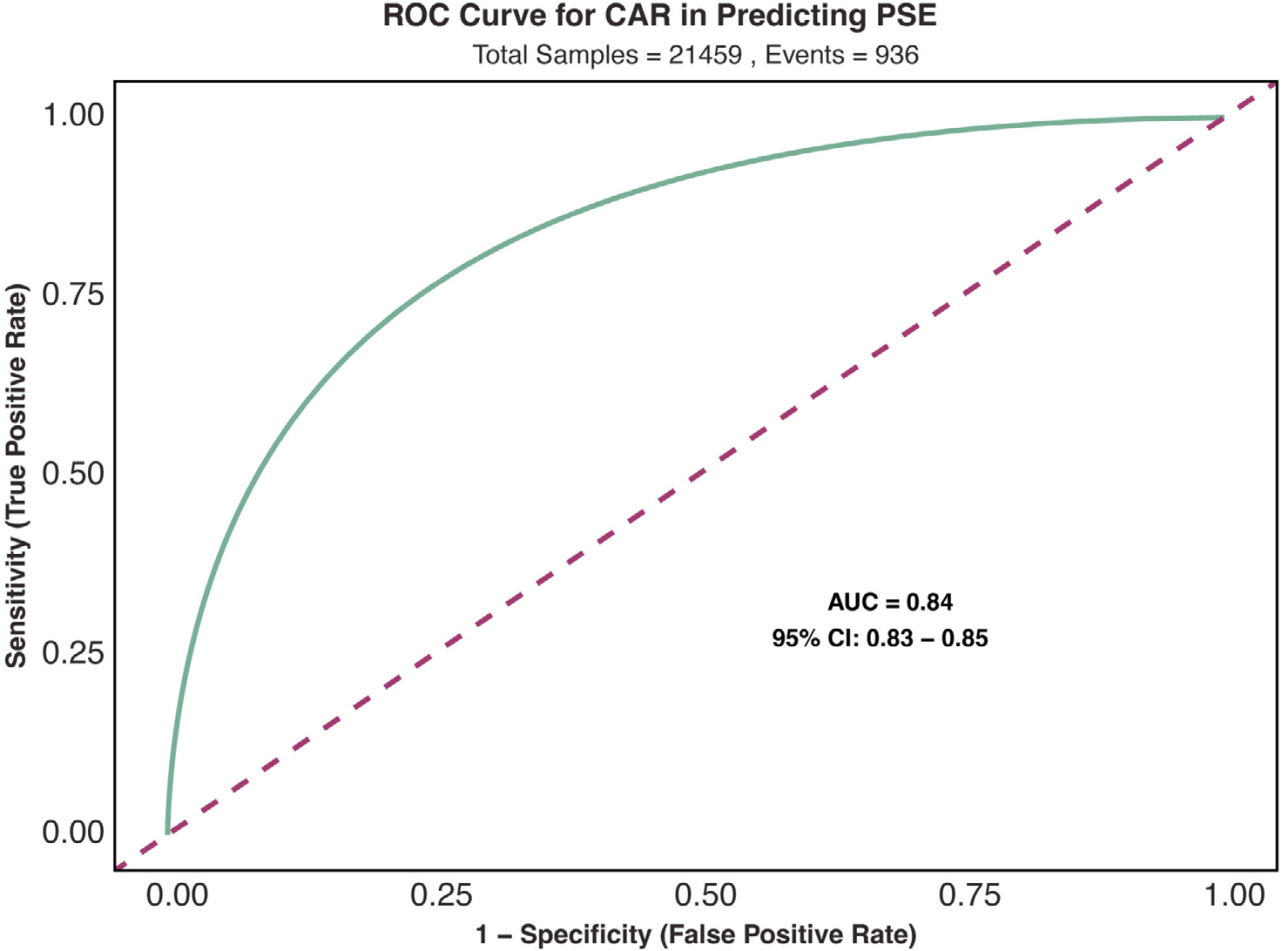
Receiver operating characteristic (ROC) analysis of CAR for predicting PSE.

In addition, to evaluate the accuracy of CAR in predicting the occurrence of PSE, we performed receiver operating characteristic (ROC) analysis, which showed an AUC of 0.84 (95% CI: 0.83–0.85) (Figure 4).

### 3.4 Subgroup Analyses

Subgroup analyses demonstrated a significant positive association between elevated CAR and increased risk of PSE (OR range: 1.69–3.07, all *p* < 0.05) across most subgroups (Table S1), including males and females, patients without uremia, DVT, fatty liver disease, diabetes, hypertension, coronary disease, atrial fibrillation, hydrocephalus, hyperuricemia, hyper lipidaemia, hypoproteinemia, and most lesion locations (frontal, parietal, temporal, insular lobes, basal ganglia, MCA territory, VA territory, CCA plaque, ICA plaque, ECA plaque, subcortical lobe, anterior and posterior circles). Significant interaction effects (P‐interaction < 0.05) were observed for several factors. Notably, the CAR‐PSE association was significantly stronger in patients without hypertension (OR = 10.27, 95% CI: 5.65–19.95) compared to those with hypertension (OR = 1.18, 95% CI: 0.97–1.44; P‐interaction < 0.001), in patients with diabetes (OR = 1.94, 95% CI: 1.56–2.42) compared to those without (OR = 1.55, 95% CI: 1.25–1.92; P‐interaction < 0.001), in patients with coronary disease (OR = 2.96, 95% CI: 2.28–3.85) versus those without (OR = 1.41, 95% CI: 1.18–1.68; P‐interaction = 0.022), and in patients without DVT (OR = 1.95, 95% CI: 1.69–2.25) versus those with DVT (OR = 1.28, 95% CI: 0.72–2.34; P‐interaction = 0.009). Neuroanatomically, a significantly stronger association was found in MCA territory lesions (OR = 7.45, 95% CI: 2.72–23.55) compared to non‐MCA lesions (OR = 1.83, 95% CI: 1.58–2.12; P‐interaction = 0.008), while a significantly reduced PSE risk was observed in occipital lobe lesions (OR = 0.14, 95% CI: 0.02–0.67) versus non‐occipital lesions (OR = 1.96, 95% CI: 1.71–2.25; P‐interaction < 0.001). Non‐significant associations were found in subgroups with uremia, DVT, hypoproteinemia, hydrocephalus, hyperuricemia, and specific lesion locations (e.g., brainstem, paraventricular, centrum semiovale), likely reflecting limited power in small subgroups or underlying biological heterogeneity.

### 3.5 Sensitivity Analyses

Four sequential sensitivity analyses were conducted to assess the robustness of the CAR‐PSE associations. These analyses included the exclusion of mild stroke cases (NIHSS < 5; adjusted OR = 1.90, 95% CI: 1.64–2.19 [Table S2]), the addition of cortical involvement adjustment (OR = 1.89, 95% CI: 1.65–2.16 [Table S3]), restriction to patients aged ≤ 80 years (OR = 1.83, 95% CI: 1.54–2.16 [Table S4]), and the analysis of carotid plaque subgroups (OR = 2.31, 95% CI: 1.90–2.81 [Table S5]). Each analysis consistently demonstrated significant associations (*p* < 0.001) and maintained dose–response gradients (Q4 vs. Q1 ORs: 24.59–59.20; all *p* < 0.001), thereby confirming the robustness of the results across key clinical strata.

## 4 Discussion

To the best of our knowledge, this study is the first to explore the relationship between CAR levels at admission and the risk of PSE. Our findings reveal a significant association between elevated CAR levels and an increased risk of PSE. Importantly, CAR demonstrated a nonlinear relationship with PSE occurrence, even after adjusting for potential confounding variables. This observation highlights the complexity of using CAR as a predictive measure for PSE risk and underscores the need for more nuanced analyses tailored to individual IS conditions. PSE poses a significant challenge in the management of IS, profoundly affecting patient outcomes and quality of life. The complexity of PSE stems from its multifaceted nature, influenced by a variety of clinical and demographic factors following IS. Understanding the predictors and underlying mechanisms of PSE is crucial for developing targeted prevention strategies and optimizing long‐term patient care.

Neuroinflammation is increasingly acknowledged as a critical factor in the pathogenesis of PSE. Inflammatory processes have been implicated in both the initiation and maintenance of epilepsy, as evidenced by studies in human and experimentally induced epilepsies [24], and more recently, in genetic epilepsies [25]. The relationship between inflammation and epilepsy is thus bidirectional, with inflammation contributing to the precipitation of seizures and seizures, in turn, inducing inflammatory responses. Following a stroke, the activation of microglia and astrocytes, along with the release of pro‐inflammatory cytokines, initiates a neuroinflammatory response that can contribute to abnormal neuronal discharges and epileptogenesis [26, 27]. Inflammatory mediators, such as cytokines and chemokines, can also directly influence neuronal excitability and network stability [28, 29]. Moreover, neuroinflammation is implicated in the development of drug‐resistant epilepsy, underscoring its role in therapeutic failures [30, 31]. Consequently, targeting neuroinflammatory pathways may represent a promising therapeutic strategy for PSE [29, 30, 32, 33]. A more comprehensive understanding of these inflammatory risk factors for PSE is essential for the development of effective treatments [34, 35].

Albumin levels serve as indicators of the body's nutritional status and protein synthesis capacity, often exhibiting a decline in the presence of chronic diseases and various inflammatory conditions. CRP, recognized as a systemic marker of inflammation, is an acute‐phase reactant synthesized in the liver, with its levels increasing in response to a range of tissue‐damaging stimuli [36]. However, CRP alone is not diagnostic and must be interpreted in conjunction with a comprehensive assessment of other clinical and pathological findings. Both CRP and albumin are synthesized in the liver and are regulated by the interleukin‐6 pathway; CRP is positively regulated, whereas albumin is negatively affected by inflammation. This differential response during inflammatory processes may explain the enhanced diagnostic utility of the combined CAR observed in previous studies [37, 38]. Although numerous cohort studies have explored the associations between various clinical and laboratory markers and the risk of PSE, definitive predictive factors have yet to be established [39]. Currently, there is limited evidence supporting the predictive value of CAR for assessing PSE risk in individuals with IS. Consequently, we conducted a comprehensive analysis using this large‐scale retrospective dataset.

In our study, which encompassed 21,459 Chinese participants diagnosed with IS, we found that individuals in the fourth quartile of baseline CAR exhibited a 34.42‐fold increased risk of PSE compared to those in the first quartile. Prior to adjusting for covariates, each unit increase in CAR was associated with a 3.36‐fold increase in PSE risk. Following comprehensive covariate adjustment, each unit increase in CAR was associated with a 1.88‐fold increase in the incidence risk of PSE. These findings indicate that even a slight elevation in CAR can significantly influence PSE risk, underscoring CAR's potential as a predictive biomarker for PSE risk, particularly in the context of preventive strategies and management within the first year following a stroke in the IS population. Furthermore, our study identified a significant nonlinear relationship between CAR and PSE events, with a threshold value of 1.15. When baseline CAR was below this threshold, a strong positive correlation with PSE risk was observed. This underscores the critical importance of monitoring CAR levels for an accurate assessment of PSE risk.

Several factors have been associated with the risk of PSE. Research has identified large stroke volume, cortical involvement, stroke severity, hemorrhagic stroke or hemorrhagic transformation, and early seizures as definitive predictors of PSE [40, 41]. A meta‐analysis encompassing 51 studies revealed that hemorrhagic transformation is significantly linked to early seizures, irrespective of whether patients with ischemic stroke undergo thrombolysis or thrombectomy [42]. Furthermore, a population‐based study involving 135,117 cases found that the risk of seizures was 0.6% for patients with an NIHSS score of less than 3 at admission, and this risk increased to 7.0% for those with a score exceeding 31 points [43]. Large vessel lesions frequently result in acute ischemic stroke, and patients who undergo mechanical thrombectomy may develop PSE as a complication. Studies suggest that larger infarct areas and the presence of cerebral microbleeds are independent predictors of PSE [44]. This suggests that in strokes caused by large vessel lesions, the severity of the lesion and the extent of brain tissue damage may directly influence the risk of epilepsy development. Thus, we performed analysis to evaluate the association between CAR and PSE in individuals with large vessel disease, or without large vessel disease, NIHSS (5–14), and NIHSS > 14. Our findings indicate a nonlinear association between CAR and PSE risk across different populations, suggesting that CAR serves as a robust predictor of PSE.

Subgroup and sensitivity analyses robustly corroborated the positive correlation between an elevated CAR and an increased risk of PSE. This association was statistically significant across the majority of pre‐specified subgroups, including those stratified by sex, various comorbidities, and most neuroanatomical lesion locations, with ORs ranging from 1.69 to 3.07. Importantly, significant interaction effects indicated that the strength of the CAR‐PSE association was modulated by specific clinical factors. The association was notably stronger in patients without hypertension and in those with diabetes or coronary artery disease. These differential associations may be attributed to the integrative nature of CAR, reflecting both inflammatory and nutritional status. In patients without hypertension, a relative lack of background inflammation might render CAR a more sensitive indicator of the acute neuroinflammatory burden post‐stroke. Conversely, in patients with diabetes or coronary artery disease, the presence of chronic systemic inflammation and metabolic dysregulation could potentially synergize with stroke‐induced neuroinflammation, amplifying the risk signal captured by an elevated CAR. Neuroanatomically, the association was significantly stronger in lesions located in the middle cerebral artery territory, whereas it was significantly attenuated in occipital lobe lesions. Furthermore, a series of sensitivity analyses, which either excluded cases of mild strokes or adjusted for additional confounders such as cortical involvement, consistently produced significant results with robust dose–response gradients. These analyses emphasize the robustness of the primary findings and indicate that the relationship between CAR and PSE is affected by particular underlying comorbidities and the topography of lesions. This highlights specific populations that may be at a disproportionately higher risk.

The CAVE score and the SeLECT score are two clinical tools used for predicting the risk of PSE. A prospective observational study in 2021 involving 915 patients with acute ischemic stroke reported that the SeLECT score predicted PSE within 1 year with an AUC of 0.756 (95% CI: 0.692–0.819) [45]. Furthermore, a 2023 systematic review and meta‐analysis evaluating several PSE prediction models, including CAVE and SeLECT, reported a pooled AUC of 0.81 (95% CI: 0.76–0.86) for the CAVE score and a pooled AUC of 0.77 (95% CI: 0.71–0.82) for the SeLECT score in predicting PSE after ischemic stroke [46]. To evaluate the predictive accuracy of the CAR for the risk of PSE within 1 year, we performed ROC analysis. The results showed an AUC of 0.84 (95% CI: 0.83–0.85). These findings suggest that CAR, as an integrated marker, holds promise for clinical translation in PSE risk assessment.

This study possesses several significant strengths. Firstly, it is grounded in a large‐scale, longitudinal cohort of IS patients, encompassing over 20,000 sample measurements, thereby ensuring robust statistical power. Secondly, data collection from inpatients adhered to consistent standards, and the association between the CAR and PSE was established through the application of multiple statistical tests, enhancing the reliability and robustness of the findings. Thirdly, these results have considerable clinical implications for the early identification of high‐risk individuals and the development of preventive strategies.

This study is subject to several limitations. Firstly, it utilized baseline CAR data; the inclusion of dynamic CAR data could potentially improve the stratification of stroke risk. Secondly, although we adjusted for multiple covariates, residual confounding from unmeasured or uncontrolled factors—such as TOAST classification, post‐stroke surgical interventions, and other potential determinants of PSE—cannot be entirely ruled out. Thirdly, a key limitation is that our data came from multiple centers with differing enrollment periods. Because individual‐level time and hospital identifiers were unavailable, we could not adjust for population incomparability. Although aggregate PSE incidence was stable (4.3%), residual bias cannot be excluded, so findings require cautious interpretation. Fourthly, we cannot precisely determine the time interval between the collection of CAR‐related biomarkers and the onset of symptoms. The levels of CRP and albumin in patients with IS undergo dynamic changes throughout the disease course, which may potentially influence the study findings. Lastly, the study population comprised Chinese adults from Chongqing city in southwestern China, which may restrict the applicability of the results to other ethnic groups.

In conclusion, this retrospective cohort study indicates that an elevated CAR at the time of admission is independently correlated with an increased risk of PSE. A nonlinear association was identified, with a threshold effect observed at a CAR value of 1.15. As an integrated marker reflecting both inflammatory and nutritional status, CAR may function as a practical and cost‐effective predictor for PSE. These findings underscore the potential of CAR to enhance risk stratification and inform early intervention strategies. Further prospective studies are necessary to validate its clinical utility.

## Author Contributions

Xiao Wu and Yingru Zhou were responsible for data analysis and manuscript preparation. Qi Yang helped to analyze the data. Yunliang Tang revised the paper and supervised the project.

## Funding

This work was supported by the Natural Science Foundation of Jiangxi Province (No. 20252BAC240573) and Clinical Research Cultivation Project of The First Affiliated Hospital of Nanchang University (No. YFYLCYJPY202526).

## Ethics Statement

Ethical approval for the present study was not required. This research is a secondary analysis of existing, de‐identified, and publicly available data from the Dryad digital repository.

## Consent

The authors have nothing to report.

## Conflicts of Interest

The authors declare no conflicts of interest.

## Supporting information


**Table S1:** Subgroup analyses of all individuals were performed to investigate the relationship between the CAR and PSE across different subgroups.
**Table S2:** Association between the CAR and PSE incidence in patients with acute ischemic stroke (NIHSS ≥ 5).
**Table S3:** Association between the CAR and PSE incidence in patients with acute ischemic stroke (further adjust for cortex involvement).
**Table S4:** Association between the CAR and PSE incidence in patients with acute ischemic stroke (age ≤ 80 years).
**Table S5:** Association between the CAR and PSE incidence in patients with acute ischemic stroke (diagnosed with carotid plaque).

## Data Availability

The data that support the findings of this study are available on request from the corresponding author. The data are not publicly available due to privacy or ethical restrictions.
