# Supplementary material for: Nonlinear Association Between the C‐Reactive Protein‐To‐Albumin Ratio and Post‐Stroke Epilepsy Risk
Source: CNS Neurosci Ther. 2026 Jul 16;32(7):e70967. doi: 10.1002/cns.70967 (PMC13374566; doi:10.1002/cns.70967)
Supplement: Supplementary file 1 — Table S1: Subgroup analyses of all individuals were performed to investigate the relationship between the CAR and PSE across different subgroups. Table S2: Association between the CAR and PSE incidence in patients with acute ischemic stroke (NIHSS ≥ 5). Table S3: Association between the CAR and PSE incidence in patients with acute ischemic stroke (further adjust for cortex involvement). Table S4: Association between the CAR and PSE incidence in patients with acute ischemic stroke (age ≤ 80 years). Table S5: Association between the CAR and PSE incidence in patients with acute ischemic stroke (diagnosed with carotid plaque). [file CNS-32-e70967-s001.docx]

| **Table S1. Subgroup analyses of all individuals were performed to investigate the relationship between the CAR and PSE across different subgroups.** | | | | | |
| --- | --- | --- | --- | --- | --- |
| Variable | Subgroup | Total (events of PSE) | OR (95% CI) | P-value | P-interaction* |
| Gender | male | 10616 (364) | 2.05 (1.58-2.67) | 0.000 | 0.004 |
|  | female | 10843 (572) | 2.05 (1.66-2.53) | 0.000 |  |
| Uremia | no | 21269 (917) | 1.90 (1.66-2.19) | 0.000 | 0.028 |
|  | yes | 190 (19) | 0.66 (0.00-221.26) | 0.889 |  |
| DVT | no | 20135 (834) | 1.95 (1.69-2.25) | 0.000 | 0.009 |
|  | yes | 1324 (102) | 1.28 (0.72-2.34) | 0.413 |  |
| Fatty liver disease or steatosis | no | 17214 (795) | 1.69 (1.46-1.97) | 0.000 | 0.246 |
|  | yes | 4245 (141) | 3.07 (2.08-4.60) | 0.000 |  |
| Diabetes | no | 14137 (607) | 1.55 (1.25-1.92) | 0.000 | 0.000 |
|  | yes | 7322 (329) | 1.94 (1.56-2.42) | 0.000 |  |
| Hypertension | no | 6708 (282) | 10.27 (5.65-19.95) | 0.000 | 0.000 |
|  | yes | 14751 (654) | 1.18 (0.97-1.44) | 0.094 |  |
| Corory_disease | no | 11787 (592) | 1.41 (1.18-1.68) | 0.000 | 0.022 |
|  | yes | 9672 (344) | 2.96 (2.28-3.85) | 0.000 |  |
| Atrial_fibrillation | no | 19416 (823) | 1.76 (1.51-2.04) | 0.000 | 0.304 |
|  | yes | 2043 (113) | 2.94 (1.79-4.94) | 0.000 |  |
| Hydrocephalus | no | 21178 (878) | 1.89 (1.64-2.17) | 0.000 | 0.753 |
|  | yes | 281 (58) | 0.18 (0.01-1.62) | 0.164 |  |
| Hyperuricemia | no | 19112 (785) | 1.88 (1.63-2.15) | 0.000 | 0.000 |
|  | yes | 2347 (151) | 8.52 (0.35-263.19) | 0.211 |  |
| Hyperlipidaemia | no | 16990 (786) | 1.90 (1.64-2.21) | 0.000 | 0.894 |
|  | yes | 4469 (150) | 1.87 (1.21-2.92) | 0.006 |  |
| Hypoproteinemia | no | 19038 (760) | 3.02 (2.55-3.58) | 0.000 | 0.000 |
|  | yes | 2421 (176) | 0.79 (0.41-1.52) | 0.480 |  |
| Frontal lobe | no | 20540 (853) | 1.85 (1.60-2.14) | 0.000 | 0.473 |
|  | yes | 919 (83) | 2.61 (1.53-4.49) | 0.000 |  |
| Parietal lobe | no | 20796 (868) | 1.82 (1.58-2.11) | 0.000 | 0.519 |
|  | yes | 663 (68) | 2.45 (1.38-4.31) | 0.002 |  |
| Temporal lobe | no | 20823 (871) | 1.84 (1.59-2.13) | 0.000 | 0.463 |
|  | yes | 636 (65) | 2.69 (1.39-5.26) | 0.003 |  |
| Occipital lobe | no | 21064 (902) | 1.96 (1.71-2.25) | 0.000 | 0.000 |
|  | yes | 395 (34) | 0.14 (0.02-0.67) | 0.022 |  |
| Insular lobe | no | 21180 (921) | 1.80 (1.57-2.07) | 0.000 | 0.345 |
|  | yes | 279 (15) | 14.07 (2.71-120.42) | 0.004 |  |
| Basal ganglia | no | 20491 (878) | 1.87 (1.61-2.16) | 0.000 | 0.389 |
|  | yes | 968 (58) | 1.91 (0.95-3.63) | 0.054 |  |
| Brainstem | no | 21187 (920) | 1.90 (1.66-2.19) | 0.000 | 0.619 |
|  | yes | 272 (16) | 0.00 (0.00-0.24) | 0.065 |  |
| Epencephalon | no | 21017 (918) | 1.92 (1.67-2.21) | 0.000 | 0.073 |
|  | yes | 442 (18) | 0.01 (0.00-0.18) | 0.005 |  |
| Paraventricular | no | 20412 (882) | 2.03 (1.76-2.35) | 0.000 | 0.000 |
|  | yes | 1047 (54) | 0.59 (0.20-1.70) | 0.338 |  |
| Centrum semiovale | no | 20849 (904) | 1.99 (1.73-2.29) | 0.000 | 0.000 |
|  | yes | 610 (32) | 0.00 (0.00-Inf) | 0.999 |  |
| Thalamus | no | 21222 (919) | 1.88 (1.64-2.16) | 0.000 | 0.527 |
|  | yes | 237 (17) | 1.45 (0.30-7.12) | 0.630 |  |
| MCA | no | 20631 (895) | 1.83 (1.58-2.12) | 0.000 | 0.008 |
|  | yes | 828 (41) | 7.45 (2.72-23.55) | 0.000 |  |
| VA | no | 20807 (909) | 1.95 (1.70-2.25) | 0.000 | 0.001 |
|  | yes | 652 (27) | 0.99 (0.30-3.69) | 0.991 |  |
| CCA plaque | no | 16636 (736) | 1.94 (1.66-2.27) | 0.000 | 0.038 |
|  | yes | 4823 (200) | 1.76 (1.27-2.43) | 0.001 |  |
| ICA plaque | no | 19997 (860) | 1.90 (1.65-2.20) | 0.000 | 0.018 |
|  | yes | 1462 (76) | 1.55 (0.79-3.04) | 0.201 |  |
| ECA plaque | no | 21258 (925) | 1.92 (1.67-2.21) | 0.000 | 0.128 |
|  | yes | 201 (11) | 4.11 (0.22-120.96) | 0.370 |  |
| Subcortical lobe | no | 19016 (801) | 1.97 (1.69-2.31) | 0.000 | 0.046 |
|  | yes | 2443 (135) | 1.49 (0.92-2.36) | 0.098 |  |
| Anterior circle | no | 20046 (868) | 1.92 (1.66-2.23) | 0.000 | 0.153 |
|  | yes | 1413 (68) | 1.58 (0.92-2.74) | 0.102 |  |
| Posterior circle | no | 16958 (762) | 1.94 (1.67-2.26) | 0.000 | 0.097 |
|  | yes | 4501 (174) | 1.62 (1.12-2.35) | 0.010 |  |
| DVT, deep vein thrombosis; ACA, Anterior cerebral artery; MCA, Middle cerebral artery; PCA, Posterior cerebral artery; VA, Vertebral artery; BA, Basilar artery; CCA plaque; Common carotid artery plaque; ICA plaque; Internal carotid artery plaque; ECA plaque, External carotid artery plaque.  *All subgroup models were adjusted for the same set of covariates as in Model 3, excluding the stratification variable itself to prevent over-adjustment. | | | | | |

| **Table S2. Association between the CAR and PSE incidence in patients with acute ischemic stroke (NIHSS≥5).** | | | | | | | |
| --- | --- | --- | --- | --- | --- | --- | --- |
| **Characteristics** | **Case/total, n** | **Model 1** | **P value** | **Model 2** | **P value** | **Model 3** | **P value** |
|  |  | **OR (95%CI)** |  | **OR (95%CI)** |  | **OR (95%CI)** |  |
| CAR (per 1 unit) | 929/19930 | 3.57 (3.33-3.83) | <0.001 | 5.55 (5.03-6.12) | <0.001 | 1.90 (1.64-2.19) | <0.001 |
| CAR quartile |  |  |  |  |  |  |  |
| Quartile 1 | 16/4983 | Reference |  | Reference |  | Reference |  |
| Quartile 2 | 36/4983 | 2.26 (1.27-4.19) | 0.007 | 3.00 (1.68-5.58) | <0.001 | 4.03 (1.80-9.92) | 0.001 |
| Quartile 3 | 169/4981 | 10.90 (6.74-18.97) | <0.001 | 17.50 (10.70-30.70) | <0.001 | 24.49 (11.68-58.02) | <0.001 |
| Quartile 4 | 708/4983 | 51.41 (32.41-88.19) | <0.001 | 102.17 (63.47-177.21) | <0.001 | 59.20 (28.51-139.10) | <0.001 |
|  | | | | | | | |

| **Table S3. Association between the CAR and PSE incidence in patients with acute ischemic stroke (further adjust for cortex involvement**). | | | | | | | |
| --- | --- | --- | --- | --- | --- | --- | --- |
| **Characteristics** | **Case/total, n** | **Model 1** | **P value** | **Model 2** | **P value** | **Model 3** | **P value** |
|  |  | **OR (95%CI)** |  | **OR (95%CI)** |  | **OR (95%CI)** |  |
| CAR (per 1 unit) | 936/21459 | 3.36 (3.14-3.59) | <0.001 | 5.22 (4.75-5.74) | <0.001 | 1.89 (1.65-2.16) | <0.001 |
| CAR quartile |  |  |  |  |  |  |  |
| Quartile 1 | 20/5365 | Reference |  | Reference |  | Reference |  |
| Quartile 2 | 26/5365 | 1.30 (0.73-2.36) | 0.377 | 1.69 (0.94-3.08) | 0.082 | 1.82 (0.86-4.01) | 0.125 |
| Quartile 3 | 166/5364 | 8.53 (5.50-14.02) | <0.001 | 12.25 (7.80-20.31) | <0.001 | 13.74 (7.32-28.14) | <0.001 |
| Quartile 4 | 724/5365 | 41.69 (27.47-67.34) | <0.001 | 73.66 (47.72-120.53) | <0.001 | 34.43 (18.58-69.75) | <0.001 |
|  | | | | | | | |

| **Table S4. Association between the CAR and PSE incidence in patients with acute ischemic stroke (age≤80 years).** | | | | | | | |
| --- | --- | --- | --- | --- | --- | --- | --- |
| **Characteristics** | **Case/total, n** | **Model 1** | **P value** | **Model 2** | **P value** | **Model 3** | **P value** |
|  |  | **OR (95%CI)** |  | **OR (95%CI)** |  | **OR (95%CI)** |  |
| CAR (per 1 unit) | 827/18439 | 3.90 (3.61-4.21) | <0.001 | 6.88 (6.14-7.72) | <0.001 | 1.83 (1.54-2.16) | <0.001 |
| CAR quartile |  |  |  |  |  |  |  |
| Quartile 1 | 14/4619 | Reference |  | Reference |  | Reference |  |
| Quartile 2 | 7/4601 | 0.50 (0.19-1.21) | 0.136 | 0.72 (0.27-1.74) | 0.481 | 1.15 (0.34-3.80) | 0.823 |
| Quartile 3 | 120/4610 | 8.79 (5.23-16.02) | <0.001 | 12.90 (7.60-23.69) | <0.001 | 19.56 (8.46-51.87) | <0.001 |
| Quartile 4 | 686/4609 | 57.52 (35.24-102.71) | <0.001 | 94.72 (57.13-171.06) | <0.001 | 54.96 (24.20-143.72) | <0.001 |
|  | | | | | | | |

| **Table S5. Association between the CAR and PSE incidence in patients with acute ischemic stroke (diagnosed with carotid plaque).** | | | | | | | |
| --- | --- | --- | --- | --- | --- | --- | --- |
| **Characteristics** | **Case/total, n** | **Model 1** | **P value** | **Model 2** | **P value** | **Model 3** | **P value** |
|  |  | **OR (95%CI)** |  | **OR (95%CI)** |  | **OR (95%CI)** |  |
| CAR (per 1 unit) | 709/16372 | 6.99 (6.23-7.88) | <0.001 | 10.03 (8.72-11.60) | <0.001 | 2.31 (1.90-2.81) | <0.001 |
| CAR quartile |  |  |  |  |  |  |  |
| Quartile 1 | 20/4096 | Reference |  | Reference |  | Reference |  |
| Quartile 2 | 6/4090 | 0.40 (0.14-0.98) | 0.058 | 0.56 (0.20-1.39) | 0.234 | 0.65 (0.19-2.05) | 0.471 |
| Quartile 3 | 94/4093 | 6.40 (3.82-11.50) | <0.001 | 9.11 (5.38-16.52) | <0.001 | 7.23 (3.38-17.21) | <0.001 |
| Quartile 4 | 594/4093 | 46.19 (28.68-80.83) | <0.001 | 73.01 (44.59-129.37) | <0.001 | 24.59 (11.74-57.61) | <0.001 |
|  | | | | | | | |
